# Coherence in the Ferroelectric A_3_ClO (A = Li, Na) Family of Electrolytes

**DOI:** 10.3390/ma14092398

**Published:** 2021-05-05

**Authors:** Maria Helena Braga

**Affiliations:** LAETA-INEGI, Engineering Physics Department, Engineering Faculty, University of Porto, R. Dr. Roberto Frias s/n, 4200-465 Porto, Portugal; mbraga@fe.up.pt

**Keywords:** decoherence, ferroelectrics, energy storage, electrolytes, van der Pol oscillators, Duffing oscillators, Poincaré maps, FitzHugh-Nagumo model, Hodgkin-Huxley model, Rabi oscillations

## Abstract

Coherence is a major caveat in quantum computing. While phonons and electrons are weakly coupled in a glass, topological insulators strongly depend on the electron-phonon coupling. Knowledge of the electron−phonon interaction at conducting surfaces is relevant from a fundamental point of view as well as for various applications, such as two-dimensional and quasi-1D superconductivity in nanotechnology. Similarly, the electron−phonon interaction plays a relevant role in other transport properties e.g., thermoelectricity, low-dimensional systems as layered Bi and Sb chalcogenides, and quasi-crystalline materials. Glass-electrolyte ferroelectric energy storage cells exhibit self-charge and self-cycling related to topological superconductivity and electron-phonon coupling; phonon coherence is therefore important. By recurring to ab initio molecular dynamics, it was demonstrated the tendency of the Li_3_ClO, Li_2.92_Ba_0.04_ClO, Na_3_ClO, and Na_2.92_Ba_0.04_ClO ferroelectric-electrolytes to keep phonon oscillation coherence for a short lapse of time in ps. Double-well energy potentials were obtained while the electrolyte systems were thermostatted in a heat bath at a constant temperature. The latter occurrences indicate ferroelectric type behavior but do not justify the coherent self-oscillations observed in all types of cells containing these families of electrolytes and, therefore, an emergent type phenomenon where the full cell works as a feedback system allowing oscillations coherence must be realized. A comparison with amorphous SiO_2_ was performed and the specific heats for the various species were calculated.

## 1. Introduction

Statistical physics relates the average behavior of a physical system with its microscopic constituents and their interactions. Under certain conditions, the equilibrium probability of a microscopic configuration x (e.g., all spin configurations, positions of all atoms, molecules, ions, etc.) is proportional to $e^{-E\left(x\right)}$, the Boltzmann distribution. The dimensionless energy $E\left(x\right)$ contains the potential energy of the system, the temperature (thermal energy kT, where T{\displaystyle T} is temperature, and k{\displaystyle k} is the Boltzmann constant), and other thermodynamic quantities [1].

Quantum mechanics offers an accurate description of a broad variety of physical systems. Nonetheless, a demonstration that quantum mechanics applies equally to macroscopic mechanical systems has been a long-abiding challenge [2].

**Figure 1:**
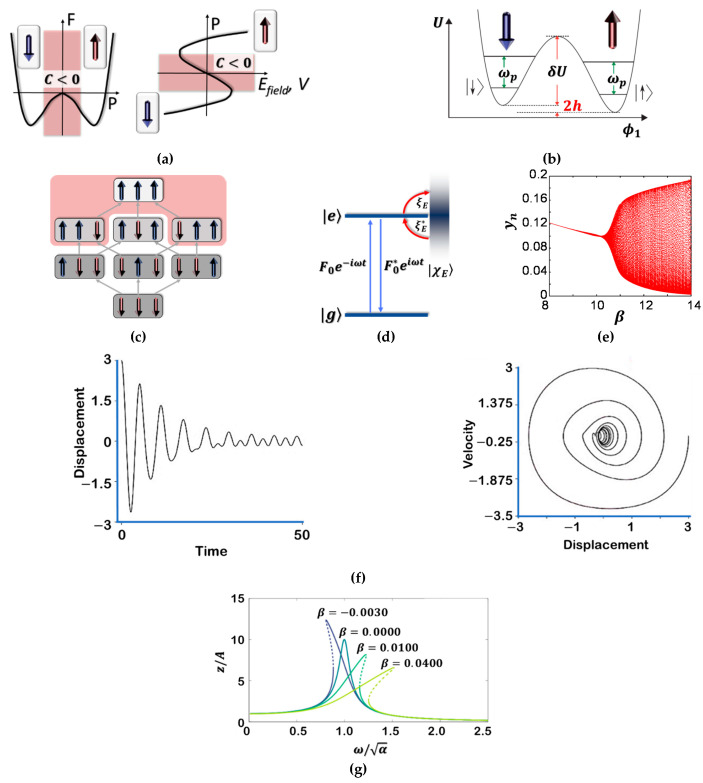
Ferroelectric, ferromagnetic, qubit, Ising model, Rabi oscillation, bifurcation, and damped-forced oscillator. (**a**) Landau–Ginzburg–Devonshire model for a ferroelectric. Left, the double-well free energy F in a ferroelectric as a function of the electric polarization P. Red shading denotes regions of negative capacitance (C < 0). Right, ferroelectric polarization–electric field / potential difference dependence obtained from the free energy F. The ‘S’-shaped P vs *E*_Field_ curve displays a region of negative capacitance related to the energy barrier of F. Arrows indicate ferroelectric polarization directions; (**b**) Double-well potential energy diagram and the lowest quantum energy levels corresponding to the qubit. States |↑ > and |↓ > are the lowest two levels, respectively. The intra-well energy spacing ω_p_. The measurement detects magnetization and does not distinguish between, |↑ > and excited states within the right-hand well. These excitations are exceedingly improbable at the time the state is measured [10]. (**c**) Minimal superatom with three spins. The configurations are coupled by spin-flips with coupling Ω (grey arrows). The interaction energy of each state is indicated by the brightness of the grey background. When selecting states with a minimal distance between spin ↑, all states in the red shaded area are neglected. While the coupling from the ground state ∣↓↓↓> to the states with n_↑_ = 1 is spatially homogeneous, the coupling of the singly excited states to the state with n_↑_ = 2 is spatially inhomogeneous [6]; (**d**) Fouquet states; model of a harmonically time-varying classical field of amplitude |F_0_| that couples the ground state |g> to the excited state |e> of a two-level system, which itself is coupled to a continuum of states |χ_E_ > [14]; (**e**) Bifurcation diagram for *y*_n_ [33]; (**f**) Duffing oscillator; displacement vs time and velocity vs displacement (phase portrait) [34]; (**g**) Frequency response z/A as a function of $\omega /\sqrt{\alpha }$ for the Duffing equation, with α=A=1 and damping δ=0.1 (Equation (1)). The dashed parts of the frequency response are unstable [35]. All the graphs cited were adapted from the reference.

Quantum Ising models (Figure 1a–c) are canonical models for the study of quantum phase transitions [3] and are the basic concept for many analog quantum computing and quantum annealing ideas [4]. Recent experiments with cold atoms have reached the interaction-dominated regime in quantum Ising magnets via optical coupling of trapped neutral atoms to Rydberg states [5]. Rydberg atoms have several unique properties including an overemphasized response to electric and magnetic fields. With the application of an external electric field, Rydberg atoms might develop exceptionally large electric dipole moments making them very susceptible to perturbation by the field [6]. Strong correlations in quantum Ising models have been observed in several experiments, starting from a single excitation in the superatom regime up to the point of crystallization. Rapid developments in this field make spin systems based on Rydberg atoms a promising platform to quantum simulation because of the unmatched flexibility and strength of interactions combined with high control and good isolation from the environment [7].

Spin Hamiltonians are repeatedly introduced as compliant simplifications of realistic condensed matter Hamiltonians and have been hugely successful in describing many materials [4,6,7,8]. Recently, they have attracted interest because they are a well-suited base for analog quantum computation (Figure 1c) [9,10,11,12,13].

Decoherence has been utilized to understand the collapse of the wave function in quantum mechanics. Specifically, components of the wave function are decoupled from a coherent system and acquire phases from their immediate surroundings. A total superposition of the global or universal wave function still exists (and remains coherent at the global level), but its fate remains an interpretational issue. Decoherence does not attempt to explain the measurement problem. Rather, decoherence explains the transition of the system to a mixture of states that seem to correspond to those states observers perceive. Decoherence embodies a challenge for the realization of quantum computers since such machines are expected to rely on the undisturbed evolution of quantum coherences. Simply stated, they require that the coherence of states be maintained and that decoherence is managed, to perform quantum computation. The preservation of coherence, and mitigation of decoherence effects, are therefore linked to the concept of quantum error correction [13].

Rabi oscillations arise when a few-level quantum system is exposed to a periodically time-varying field Figure 1d [14]. These oscillations are pervasive in many areas of physics, from atomic [15,16] and condensed-matter physics to quantum information science, having been reported for Rydberg atoms [17], nuclear spin states of different exchange symmetry [18], cavity polaritons [19,20,21], impurity states in insulators [22], excitons [23], and spin states in quantum dots [24,25] as well as Josephson junctions [26]. Floquet states (Figure 1d) are stationary solutions of a quantum system in the subjected to a periodic time-dependent perturbation. Such states have enticed considerable attention recently, arising primarily from the possibility that new phases with interesting topological properties may emerge under high-intensity excitation [26,27,28,29,30,31,32].

If a system’s parameter is gradually varied, it produces gradual variations in the state of the system itself. There can be circumstances, however, in which gradual variations of a parameter cause sudden or catastrophic changes of state. A system of this kind shows bifurcations [10] (Figure 1e). Bifurcation theory has been implemented to correlate quantum systems to the dynamics of their classical analogs in atomic systems [33,34,35,36,37,38], molecular systems [39], and resonant tunneling diodes [40]. Bifurcation theory has also been applied to the study of laser dynamics [41], and many theoretical examples, which are hard to reach experimentally such as the kicked top [42] and coupled quantum wells [43]. The leading reason for the link between quantum systems and bifurcations in the classical equations of motion is that at bifurcations, the mark of classical orbits becomes large [44,45]. Many kinds of bifurcations have been studied with respect to the associations between classical and quantum dynamics including saddle node bifurcations, Hopf bifurcations, umbilic bifurcations, period doubling bifurcations, reconnection bifurcations, tangent bifurcations, and cusp bifurcations [45].

One of the dynamical systems exhibiting chaos is the non-linear forced oscillator:

(1) $$\ddot{x}+\delta \dot{x}+\alpha x+\beta x^{3}=A\;cos\omega t$$

where x is an extensive variable such as the displacement or the charge, δ is the damping parameter, α controls the linear stiffness, β controls the amount of non-linearity in the restoring force; if β=0, the Duffing equation describes a damped and driven simple harmonic oscillator, and f=A cosωt is the driving term, A is the amplitude of the periodic driving force and ω the angular frequency. If β≠0, the oscillator is a forced-damped Duffing oscillator (e.g., Figure 1f,g) [46,47].

Nonlinearity does not always act as a vehicle of disorder or chaos. In fact, it can have exactly the opposite effect; an example of that, are van der Pol’s self-oscillations with limit cycles.

The *relaxation oscillations* have become the foundation of geometric singular perturbation theory and play a significant role in the analysis presented here. The circuit showing relaxation oscillations consists of a *feedback loop* containing a switching device such as a transistor, comparator, relay [48], or a negative resistance device like a tunnel diode, that repetitively charges a capacitor or an inductor through a resistance until it reaches a threshold level and then discharges again [49,50]. The period of the oscillator depends on the time constant of the capacitor and/or inductor circuit [51]. The active device switches abruptly between charging and discharging modes and thus produces a discontinuously changing repetitive waveform [49,51,52].

**Figure 2:**
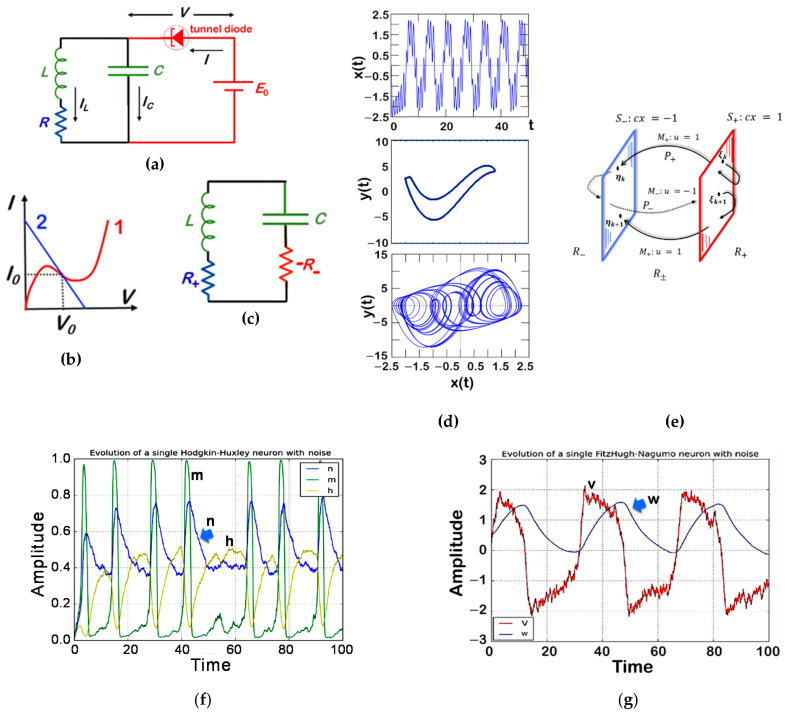
Van der Pol oscillator; (**a**) electrical circuit of a van der Pol oscillator with a tunnel diode showing negative resistance; (**b**) I vs V characteristic $I=f\left(V\right)$ of the tunnel diode (curve 1) and load line $I=\left(E_{0}–V\right)/R$ for a constant current (line 2). These two lines intersect at the operation point $\;V=V_{0}$, $I=I_{0}$; (**c**) equivalent circuit of the tunnel-diode oscillator for alternating current with a negative resistor –R**_-_**(adapted from [63]); (**d**) the x vs time series of Equations (2) and (3), Poincaré map, and phase portrait (projection of the extended 3D portrait) for values A = −50, ε = 5 and ω = 7, corresponding to almost periodic orbit (obtained in this study using wxMaxima 20.06.6 and the Runge-Kutta method, supplementary information) - Poincaré map adapted from [52]; (**e**) partitioning of the state space and the Poincaré maps [48]; (**f**) noisy Hodgkin-Huxley model; time evolution of the three ion channel variables n, m, and h [64]; (**g**) time evolution of the membrane potential and the adaptation variable in the noisy FitzHugh-Nagumo model (V—voltage, w—current). (**f,g**) adapted from [64].

Van der Pol proposed a version of a relaxation oscillations circuit that includes a periodic forcing term (Figure 2):

(2) $$\ddot{x}+\varepsilon \left(x^{2}-1\right)\dot{x}+x=A\;sin\;\omega t$$

Equation (2) can be written as:

(3) $$\left\{\begin{array}{c} \dot{x}=y\;\\ \dot{y}=-x+\varepsilon \left(1-x^{2}\right)y+A\cos\left(\omega t+\varphi \right) \end{array}\right.$$

In equations such as Equations (2) and (3), van der Pol and van der Mark [53] noted the existence of two stable periodic solutions with different periods for a particular value of the parameters and observed noisy behavior in an electrical circuit described by Equation (2) [53]. In their investigations of the oscillator behavior in the relaxation oscillations regime, they found that the subharmonic oscillations could be observed during changes in the natural frequency of the system [52]. Moreover, the authors noted “irregular noise” before the transition from one subharmonic regime to another. Their paper is perhaps one of the first experimental reports of chaos-which they did not engage in in more detail [52,53].

Since its introduction in the 1920s, the van der Pol equation has been studied for systems with self-excited limit cycle oscillations. The classical experimental setup of the system is the oscillator with a vacuum triode. The equation has been studied over wide parameter regimes, from perturbations of harmonic motion to relaxation oscillations. The Van der Pol equation is now used as a basic model for oscillatory processes in physics, electronics, biology, neurology, sociology, and economics [54,55,56,57,58,59]. Van der Pol himself built some electronic circuit models of the human heart to study the range of stability of heart dynamics. His investigations with adding an external driving signal were like the situation in which a real heart is driven by a pacemaker. Van der Pol was interested in finding out how to stabilize a heart’s irregular beating or “arrhythmias”. Since then, his equivalent circuit has been used by scientists to model a variety of physical and biological phenomena. For instance, in biology, the van der Pol equation has been used as the basis of a model of a couple of neurons in the gastric mill circuit of the stomatogastric ganglion [60,61]. The Hodgkin-Huxley and FitzHugh-Nagumo equation is a planar vector field that extends the van der Pol equation as a model for action potentials of neurons (Figure 2f,g). In seismology, the van der Pol equation has been utilized in the development of the model of the interaction of two plates in a geological fault [62].

## 2. Contextualizing to the Ferroelectric Glass-Electrolyte Cells

In recent years, we have been observing different manifestations of the van der Pol-Duffing Hodgkin-Huxley and FitzHugh-Nagumo oscillators, in different architectures of electrochemical/electrostatic energy harvesting and storage cells [65,66,67,68,69,70,71]. We have studied what seems to be analogous to Rabi’s oscillations arising from Floquet states, namely, stationary solutions of a quantum system in the presence of a periodic time-dependent perturbation [70].

The self-charge, leading to an increase in potential difference and current [70,71,72,73,74], while imparting energy to an electrical load resulting in an mA discharge current, is novel in the energy storage world.

The only way the voltage of an electrochemical cell can increase is if the chemical potential µ of the negative electrode increases, the positive decreases, or both (µ_vaccum_ = 0 eV). A cell’s voltage upsurge is only accomplished if electrons are conducted from the positive electrode to the negative electrode, which is the charging direction. As the cell is discharging, the charging current is internal to the cell completing the feedback, as in the Poincaré maps of Figure 2d,e. Since the electrolyte is an insulator and the electrical double-layer capacitors at the interfaces are maintained, the current seems to be topological and the alkali mobile ions in the bulk electrolyte do not cease to move towards the positive electrode while discharging. Surface electrons tunnel from the electrolyte to the negative electrode completing the Poincaré maps. At the negative electrode, Rabi oscillations like those shown in Figure 1d take place.

We concluded that a topologic supercurrent can be feedback in a ferroelectric-electrolyte cell; self-charging materializes by the tunneling of electrons from the electrolytes’ surface to the negative electrode while the cell is discharging with an external load (such as material resistors or LEDs) [70,71,72,73,74]. The negative electrode/ferroelectric electrolyte/positive electrode cells are then able to maintain self-sustained oscillations above a certain voltage for days, like those described by the van der Pol and Fitz Hugh-Nagumo models [70,71]. Phenomena observed in these ferroelectric-electrolyte cells are similar to those in Figure 2f,g and the physical feedback occurring in the cells can be described as the Poincaré maps of Figure 2e; a trajectory that has at least a finite number of switchings. Let $\xi_{k}$ be the point on the trajectory where it switches to $M_{+}$ i.e., $\xi_{k}\in S_{+}$. Suppose that after time $t_{k}$, the trajectory hits $S_{-}$ at $\eta_{k}$ and switches to $M_{-}$. Let $\tau_{k}$, be the time taken for the trajectory to hit $S_{+}$ again at $\xi_{k+1}$. Suppose that the trajectory is transversal to the switching surfaces at $\xi_{k}\;$ and $\eta_{k}$. Two Poincaré maps $\eta_{k}=P_{+}(\xi_{k}$) and $\xi_{k+1}=P_{-}(\eta_{k}$) are then defined. The composition $P=P_{-}\circ P_{+}$, which maps $S_{+}$ to itself, defines a Poincaré return map for the feedback system. Similarly $\tilde{P}=P_{+}\circ P_{-}$, which maps $S_{-}$ to itself, is also a Poincaré return map (Figure 2e). The fixed points and periodic points of a return map correspond to the periodic orbits of the feedback system [48].

**Figure 3:**
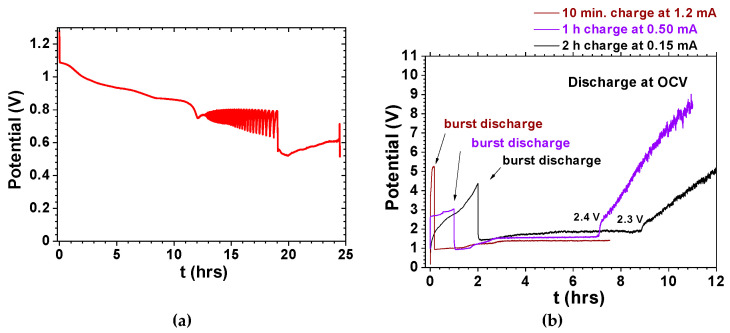
Conductor_1_/ferroelectric-electrolyte/conductor_2_ cells showing self-charging and self-cycling while the cell is set to discharge. (**a**) Zn/ferroelectric-electrolyte (Na_2.99_Ba_0.005_ClO)/C + Cu cell showing bifurcations corresponding to self-cycling while discharging with a resistor of 1000 Ω at 60 °C as in Figure 1e (adapted from [70]); (**b**) Cu/ferroelectric-electrolyte (Na_2.99_Ba_0.005_ClO)/Cu cell after quick charge followed by discharge at open-circuit voltage to 1.1–1.3 V, for then self-charging to 2.3–2.4 V after 6 or 8 h up to approximately 9 or 5 V, respectively (adapted from [65]).

**Figure 4:**
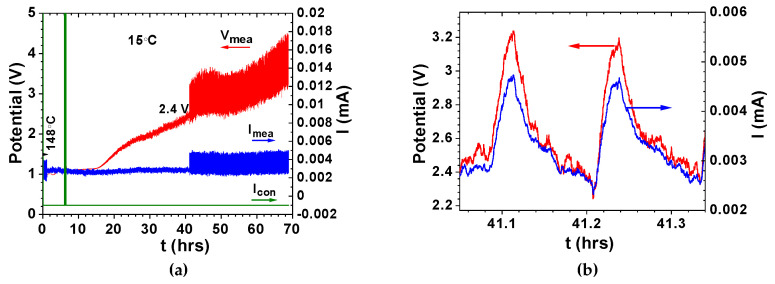

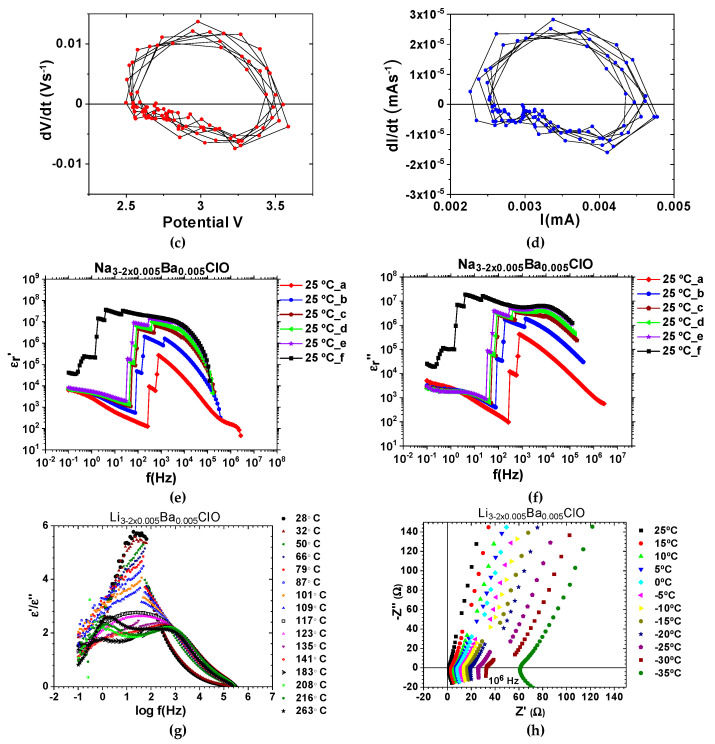
Conductor_1_/ferroelectric-electrolyte/conductor_2_ cells showing self-charging and self-cycling while set to discharge and amplitude jumps (discontinuities) related frequency response in a Duffing oscillator as shown in Figure 1g; (**a**) self-charge and self-oscillations observed with an Al/90 wt. % Li-glass (Li_2.99_Ba_0.005_ClO) + 10 wt. % Li_2_S/Cu cell while set to discharge at a constant current of −1 µA (adapted from [70]); (**b**) inset of (**a**) showing small oscillations like n in Figure 2f and w (current) in Figure 2g; (**c**,**d**) phase portraits of the voltage and current oscillations shown in (**a,b**) which clearly show limit cycles; (**e**,**f**) relative real and imaginary permittivities for Na_2.99_Ba_0.005_ClO for different times at 25 °C showing jumps in the amplitude of the forced oscillations imposed by the electrical impedance spectroscopy (EIS) measurement (adapted from [66]); (**g**) relative real and imaginary permittivities ratio for Li_2.99_Ba_0.005_ClO, at different temperatures, showing jumps in the amplitude of the forced oscillations imposed by the electrical impedance spectroscopy (EIS) measurement driving forces; (**h**) imaginary vs real impedances for Li_2.99_Ba_0.005_ClO, at different temperatures, showing jumps in the amplitude of the forced oscillations imposed by the electrical impedance spectroscopy (EIS) (adapted from [64]). Note: EIS is an AC technique, therefore, a frequency response as shown in Figure 1g for a Duffing oscillator is expected. The voltage amplitude was 10 mV.

In the material ferroelectric-electrolyte cells, the $S_{-}$ Poincaré section is the negative electrode and the $S_{+}$ is the positive electrode. $\xi_{k}$ is the point on the trajectory where an electron tunnels from the positive electrode to the surface of the ferroelectric-electrolyte $M_{+}$ where it is freely conducted (as in a topological superconductor) reaching $S_{-}$ after tunneling from the surface of the electrolyte at $\eta_{k},$ after $t_{k}$. The trajectory of the electron then switches to $M_{-}$ which is the external circuit during discharge; for example, a circuit constituted by a load resistor which is connected to the ferroelectric-electrolyte cell where the electron is conducted back to $S_{+}$ at $\xi_{k+1}$. Then the electron tunnels back to $M_{+}$ where it is conducted through the surface of the ferroelectric to tunnel back to $\eta_{k+1}$ at $S_{-}$ (the negative electrode) a Poincaré return map for the feedback cell corresponding to the experimental phenomena occurring in Figure 3 and Figure 4 [70,71].

In our previous studies, it was demonstrated that coherence might be observed while discharging electrostatic/electrochemical cells with a load [65,66,67,68,69,70,71,72,73,74]. In other words, the cells of the type conductor_1_/ferroelectric-electrolyte/conductor_2_ with collective electrical self-charge and self-cycling phenomena look like a single macroscopic quantum object while discharging with a load, as observed in Figure 3 and Figure 4. The ferroelectric-electrolytes (Na_2.99_Ba_0.005_ClO and Li_2.99_Ba_0.005_ClO) and their polymer composites have huge dielectric constants such as those observed in Figure 4e–h; the conductor-electrodes in conductor_1_/ferroelectric-electrolyte/conductor_2_ do not contain sodium or lithium, in any form, when the cells are assembled. Experiments show that even when discharge is set at relatively high currents such as 0.4–0.2 mA cm^−2^, coherent self-charge and self-cycling can be observed, especially above 35 °C [70,71,72,73,74].

As in the Poincaré maps where a periodic solution of the feedback system is attained (Figure 2d,e), it is possible to achieve a periodic self-charge/discharge in cells of the type conductor_1_/ferroelectric-electrolyte/conductor_2_, as shown in Figure 3 and Figure 4a–d [65,66,67,68,69,70,71,72,73,74]. Results obtained with Na_2.99_Ba_0.005_ClO and Li_2.99_Ba_0.005_ClO ferroelectric-based cells show that self-charge and self-cycles may be described with van der Pol’s model (Figure 3 and Figure 4). In certain circumstances, the voltage of our cells can be described by Duffing oscillators as, for example, in Figure 4e–h similar to Figure 1g.

This study aimed to observe the relationship between the microscopic and macroscopic properties of the amorphous simulated Li_3_ClO, Na_3_ClO, Li_2.92_Ba_0.04_ClO, Na_2.92_Ba_0.04_ClO electrolytes, and to compare them with the amorphous SiO_2_. Understanding if the latter may withstand decoherence, or if full-cell emergent phenomena are necessary to observe coherence, was an additional goal. It is noteworthy that the features obtained hereafter in this study for the latter ferroelectric-electrolytes are related to phonons, not with the electrons. These behaviors are nonetheless linked. As in superconducting topological behavior, the electron-phonon coupling is observed.

To what extent are the configurations of the ferroelectric-electrolyte cells propitiating coherent self-oscillations (such as those in Figure 3 and Figure 4)? Is it a ferroelectric-electrolyte solely driven phenomenon? Or it requires a certain cell architecture? What is the role of the electrolytes’ Barium in decoherence? The goal of this study is to shed light on these questions.

## 3. Theoretical Methods

The relationship between microscopic and macroscopic properties is not intuitive, because fluctuations of properties are significant in microscopic systems. It is required, therefore, to collect multiple snapshots of a microscopic system to compute a meaningful average property. This collection of snapshots is a statistical ensemble.

The molecular dynamics (MD) method enables one to build of a model in which the molecules are continuously moving. It simulates the behavior of a limited number of molecules, confined to a given volume and interacting with one another through a given pair potential [75]. The molecules are endowed with more or less ordered position coordinates at the start, so that none overlap, but with random coordinates of velocity. Their trajectories will then be followed, by a detailed solution of Newton’s laws of motion.

In an ab-initio molecular dynamics (AMD) run the forces calculated in a given geometry step are used to update the atomic positions. The system dynamics, i.e., the ionic movements, are subject to Newton mechanics while the forces acting on the ions are calculated from ab initio using a self-consistent electronic density (Hellmann-Feynman forces) as implemented in Vienna Ab Initio Software Package (VASP) [76].

Periodic boundary conditions are assumed and allow that a molecule, which leaves the volume across one face, re-enters across the opposite phase.

In the statistical ensemble, the various states of the system differ in positions and velocities of the component’s particles. The space of all possible system states is of dimension 6N for N particles and is termed the phase space.

A system at imposed volume  V’, and temperature T, is represented by a statistical ensemble, which is called the canonical ensemble or $NV^{\prime }T$ ensemble.

The number of molecules and the global volume are identical for all system states belonging to the ensemble, but they differ in total energy, which is a fluctuating variable in this ensemble. Each state *j* of the canonical ensemble occurs with a probability proportional to $e^{-E_{j}/k_{B}T}$ where $k_{B}$ is the Boltzmann and $E_{j}$ is the total energy (kinetic + potential) of the system in state *j*. Here we use the symbol E for total energy (kinetic and potential) and U for the potential energy. The Boltzmann factor $e^{-E_{j}/k_{B}T}$ expresses that low energy states are favored when compared to high-energy states. Increasing temperature broadens the energy distribution in the ensemble and, as a consequence, the average energy is increased.

Ab initio molecular dynamics (AMD) as implemented by VASP [76] was used to simulate a closed system thermostatted in a heat bath at a constant temperature as in an $NV^{\prime }T$ ensemble. The studied systems were Li_81_Cl_27_O_27_, Li_79_BaCl_27_O_27_, Na_81_Cl_27_O_27_, Na_79_BaCl_27_O_27_, Si_3_O_6_, and Si_108_O_216_ where the compositions of the compounds containing barium, were the most approximate to A_2.99_Ba_0.005_ClO (A = Li or Na), while still maintaining a feasible simulating computing time.

The initial structures were optimized at the correspondent temperatures after performing microcanonical simulation *NV’E* and isothermal-isobaric simulations *NP’T* on the crystalline optimized structure to set the volumes at the correspondent temperatures. The temperatures were chosen to assure that the system is in the amorphous state, immediately above the transition to amorphous.

**Figure 5:**
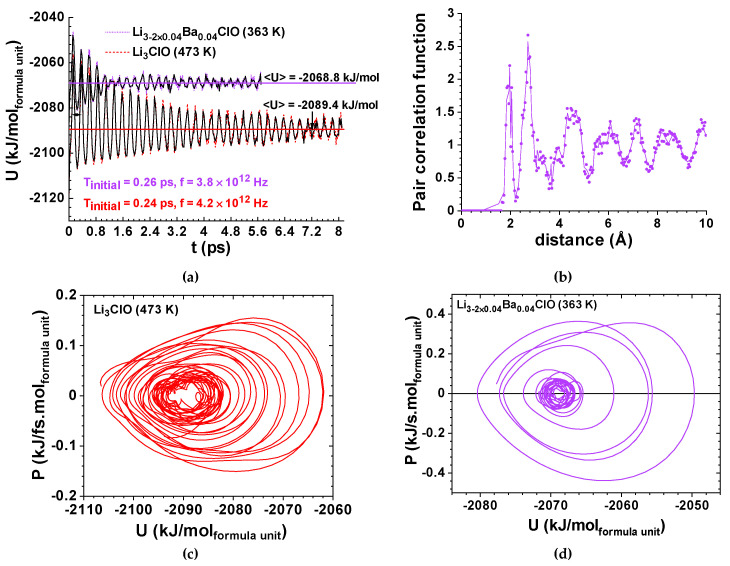
Ab Initio Molecular Dynamics for Li-glass ferroelectric-electrolytes; (**a**) potential energy vs time for Li_2.92_Ba_0.04_ClO and Li_3_ClO. The upper envelope energy for Li_2.92_Ba_0.04_ClO at 363 K before decoherence: $-2085+41.94e^{-t/5.634}$ kJ/mol_formula unit_ corresponding to a damping oscillator; decoherence is observed above 1.2 ps; the calculated specific heat is C_v’_ = 0.193 kJ/kg K; the upper envelope energy for Li_3_ClO at 473 K before decoherence:$-2085+30.23e^{-t/2.012}$ kJ/mol_formula unit_ corresponding to a damping oscillator; decoherence is observed above 7 ps; the calculated specific heat is C_v’_ = 0.465 kJ/kg K; (**b**) pair correlation function for Li_2.92_Ba_0.04_ClO, at 363 K, showing first and second neighbors at fixed distances corresponding to sharp peaks and broad peaks for third and above neighbors’ distances indicating structural disorder, often associated with an amorphous character, as expected from experimental results; (**c**) phase portrait for power vs. energy for Li_3_ClO at 473 K corresponding to a damped coherent oscillator that becomes decoherent; the relaxation of the focus (equilibrium energy) corresponds to a variation of 0.3% from the initial focus energy; ΔU_max_ = U_max_ − U_min_ ≈ 0.46 eV; (**d**) phase portrait for power vs energy for Li_2.92_Ba_0.04_ClO, at 363 K, corresponding to a damped coherent oscillator that becomes decoherent; the relaxation of the focus corresponds to a variation of 0.2% of the initial energy; ΔU_max_ ≈ 0.32 eV. Note: both (**b**) and (**c**) are similar to the Duffing oscillator model in Figure 1f.

**Figure 6:**
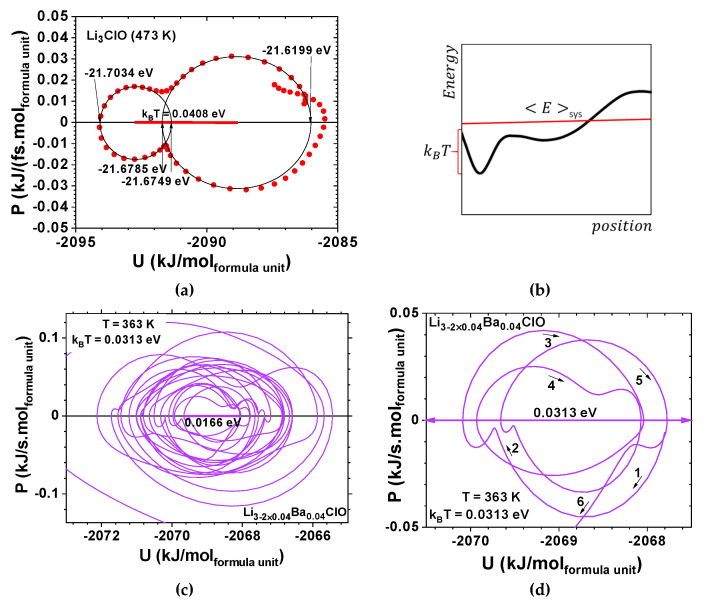
Ab Initio Molecular Dynamics for Li-glass ferroelectric-electrolytes showing decoherence; (**a**) inset of the phase portrait of Li_3_ClO showing a double-well potential; (**b**) schematic representation of energy vs position, showing that thermal excitation allows a molecule to hop back and forth between the wells; (**c**,**d**) insets of the phase portrait of Li_2.92_Ba_0.04_ClO showing back and forth paths between the wells described by a van der Pol-Duffing oscillator model as the one in Figure 1f and Figure 2d. Note: Potential energy is dU=−F.dr and power is dP=F.dv where F is an applied force, r atom displacement, and v atom velocity.

**Figure 7:**
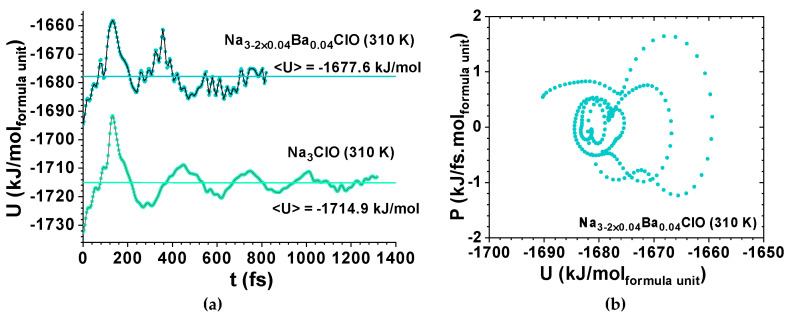
Ab Initio Molecular Dynamics for Na-glass ferroelectric-electrolyte showing decoherence; (**a**) potential energy vs time for Na_2.92_Ba_0.04_ClO at 310 K; the calculated specific heat is C_v’_ = 0.442 kJ/kg K and potential energy vs time for Na_3_ClO at 310 K; the calculated specific heat is C_v’_ = 0.457 kJ/kg K; (**b**) phase portrait of Na_2.92_Ba_0.04_ClO for power vs potential energy showing back and forth between two double-wells described by a van der Pol-Duffing oscillator model as the one in Figure 1f.

**Figure 8:**
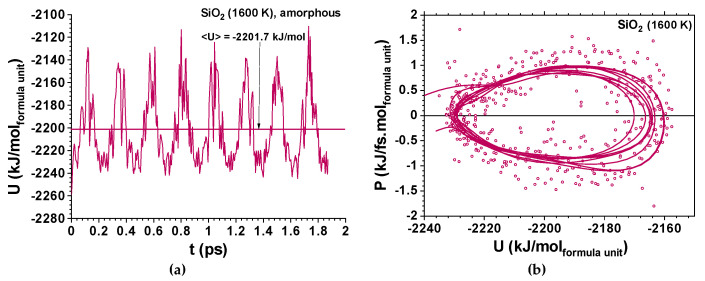
Ab Initio Molecular Dynamics for the amorphous Si_3_O_6_ showing noise and a quasi-periodic structure; (**a**) total energy vs time for SiO_2_ at 1600 K; the calculated specific heat is C_v’_ = 0.826 kJ/kg K; no damping is observed; (**b**) phase portrait of SiO_2_ for power vs potential energy showing one energy well and almost no anharmonicity. The difference between the two energy states is ΔU ≈ 0.62 eV and k_B_T = 0.129 eV. Almost no focus (equilibrium energy) displacement is observed. Note that simulations not shown here demonstrate that for a larger box such as that containing Si_108_O_216_, decoherence is achieved immediately even at room temperature.

Projector augmented wave (PAW) potentials [77] in the generalized gradient approximation (GGA) [78] were used. Electronic structure calculations were conducted with cut-off energies of 500 eV. The AMD simulations were performed with 4 fs time-steps to a maximum of 8 ps; velocities were rescaled to maintain a constant temperature using the Nosé’s thermostat [79] with a mass chosen such that temperature fluctuates with a period of 40 time-steps. Each NV’T ensemble was allowed to relax at least until decoherence was noticeably observed. Results are shown in Figure 5, Figure 6, Figure 7 and Figure 8.

A finite collection of system states, which is representative of the $NV^{\prime }T$ statistical ensemble, average properties was obtained by simple arithmetic averaging over the n states composing the collection. For instance, the average energy is $\langle E\rangle =\frac{1}{n}\overset{n}{\underset{i=1}{\sum }}E_{i}$. The heat capacity at constant volume is $C_{V^{\prime }}={\left(\frac{\partial E}{\partial T}\right)}_{V^{\prime }}=\frac{1}{k_{B}T^{2}}\left(\langle E^{2}\rangle -{\langle E\rangle }^{2}\right)$.

The pair distribution functions $g\left(r\right)$ versus the interatomic distances r were also calculated for controlling the non-periodic character of the amorphous structures. An example of the latter is shown for Li_2.92_Ba_0.04_ClO in Figure 5b.

It is important highlighting that if a manifestation is observed during the AMD relaxation, the correspondent event should be witnessed in Nature. Nonetheless, processes with slow dynamics will not be observed in AMD, even if occurring in Nature.

The *x* vs time series and the phase portrait (projection of the extended 3D portrait) for values A = −50, ε = 5, and ω = 7, corresponding to almost periodic orbit in Figure 2d were obtained using wxMaxima 20.06.6 and the Runge-Kutta method in supplementary information.

## 4. Results and Discussion

The results obtained for the ferroelectric amorphous-electrolytes have been demonstrated to be very different from those obtained for the amorphous SiO_2_ that was used as a control, as its heat capacity is well known (Figure 5, Figure 6, Figure 7 and Figure 8).

While Li_3_ClO phonons’ oscillations corresponded to a strong environmental coupling between 0 and 7.0 ps, Li_2.92_Ba_0.04_ClO phonon oscillations were only strongly coupled up to 1.2 ps (Figure 5). Conversely, Si_3_O_6_ shows an almost periodic orbit (Figure 2d) where noise is observed since t=0 ps. As expected, Na_3_ClO maintains a certain coherence during the first ps, while Na_2.92_Ba_0.04_ClO phonon oscillations show decoherence almost immediately (Figure 7).

Whereas Li_3_ClO and Li_2.92_Ba_0.04_ClO show single-well oscillations in the first ps (Figure 5 and Figure 6), Na_3_ClO and Na_2.92_Ba_0.04_ClO show almost instantaneous decoherence while their energy oscillates in a double-well (Figure 7). Si_3_O_6_ energy oscillates in a single well and shows noise since the first time-steps (Figure 8). Control simulations performed with Si_108_O_216_ show that SiO_2_ phonon oscillations are immediately decoherent (not showing any periodicity), even at room temperature, which indicates that if the simulation box contains enough atoms, the decoherence is obtained faster. It is not trivial to obtain a canonical ensemble distribution of conformations and velocities as they depend on system size, thermostat, and time step.

Although decoherence is no doubtfully more prominent at elevated temperatures due to its very nature, in this study it was observed that the substitution of two Lithium atoms by a heavy Barium atom and a vacancy is more important, surpassing the effect of temperature as observed in Figure 5; Li_3_ClO keeps its coherence for a much longer period than Li_2.92_Ba_0.04_ClO, even if its temperature is 110 K higher. The same applies to the Na-based ferroelectric electrolyte as shown later. We had previously observed experimentally that the introduction of a small amount of Ba in Li_2.99_Ba_0.005_ClO reduces the glass transition temperature of Li_3_ClO.

Two very distinctive features are found when comparing the amorphous ferroelectric electrolytes and the amorphous Si_3_O_6_ AMD relaxation oscillations; while the first is dissipative and anharmonic (Figure 5), in the second the dissipation is not observed and the system keeps its focal energy point fairly well; no exponential dissipation envelop-type could be observed in Figure 8 for SiO_2_ at 1600 K. This phenomenon does not seem to be directly related with the temperature difference as simulations for the latter at 298 K show comparable results.

The upper envelope of the potential energy for Li_2.92_Ba_0.04_ClO, at 363 K, before decoherence, is −2085 + 41.94e^-t/5.634^ kJ/mol_formula unit_ corresponding to a damping oscillator. Decoherence is observed above 1.2 ps; the calculated specific heat at constant volume is C_v’_ = 0.193 kJ/kg K. The upper envelope energy for Li_3_ClO at 473 K before decoherence is −2085 + 30.23e^-t/2.012^ kJ/mol_formula unit_ corresponding to a damping oscillator; decoherence is observed above 7.0 ps (Figure 5). The calculated specific heat at constant volume is C_v’_ = 0.465 kJ/kg K. The difference between envelope curves should be essentially related to Ba doping.

The total energy vs time for Na_2.92_Ba_0.04_ClO, at 310 K, shows almost instantaneous decoherence; the calculated specific heat at constant volume is C_v_’ = 0.442 kJ/kg K (Figure 7). For Na_3_ClO at 310 K the decoherence occurs at approximately 1 ps and the calculated specific heat at constant volume is C_v_’ = 0.457 kJ/kg K (Figure 7). For SiO_2_ at 1600 K, the calculated specific heat at constant volume is C_v_’ = 0.826 kJ/kg K (Figure 8).

## 5. Conclusions

The ab initio molecular dynamics canonical ensemble was used to describe the thermodynamics of five different amorphous systems (Li_3_ClO, Li_3−2__× 0.04_Ba_0.04_ClO, Na_3_ClO, Na_3-2__× 0.04_Ba_0.04_ClO, and SiO_2_) as the systems were thermostatted in a heat bath at a constant temperature. This procedure allowed calculating the potential energy and the average of the total energy of each system, as well as, the specific heat at constant volume. The results for Li_3_ClO, Li_3−2__× 0.04_Ba_0.04_ClO, Na_3_ClO, and Na_3−2__× 0.04_Ba_0.04_ClO specific heat at constant volume are in agreement with the experimental results of the end binaries; for SiO_2_ the calculated specific heat is within the experimental error of data found in the literature.

The ferroelectric nature of the Li_3_ClO, Li_3−2 × 0.04_Ba_0.04_ClO, Na_3_ClO, and Na_3−2__× 0.04_Ba_0.04_ClO was observed as the potential energy relaxed to two energy minima accessible by thermal energy, k_B_T, corresponding to symmetric configurations of dipoles or dipoles coalescences as in an Ising model. These systems tend to be described by van der Pol– Duffing oscillators.

In this study, it was observed that the substitution of two lithium or sodium atoms by a heavy barium and a vacancy is more important than the effect of temperature in decoherence.

Although the ferroelectric character of the electrolytes, the van der Pol–Duffing relaxation-oscillator models and a certain coherence are undoubtedly observed for the simulated electrolytes, at the temperatures at which the conductor_1_/ferroelectric-electrolyte/conductor_2_ cells were observed to self-charge and self-cycle; the time-scales for coherence observation are much smaller. Therefore, it is likely that a cell containing two electrodes with different electrochemical potentials and the electrolyte is needed to have relaxation oscillations in a timescale of hours or days confirming the phenomenon as emergent. The external circuit discharge with the internal self-charge feedback enabled by the coupled electron-phonon surface conduction of the ferroelectric-electrolyte constitutes two Poincaré maps. This latter feedback described by equivalent circuits such as van der Pol and Hodgkin-Huxley FitzHugh-Nagumo is likely to constitute a necessary condition to achieve coherence in a ferroelectric-electrolyte cell.

## Data Availability

The data is available upon demand.

## Supplementary Materials

The following are available online at https://www.mdpi.com/article/10.3390/ma14092398/s1.
