# Supplementary material for: Coherence in the Ferroelectric A3ClO (A = Li, Na) Family of Electrolytes
Source: Materials (Basel). 2021 May 5;14(9):2398. doi: 10.3390/ma14092398 (PMC8125208; doi:10.3390/ma14092398)
Supplement: Supplementary file 1 [file materials-14-02398-s001.zip › materials-1163549-SI.pdf]

materials

Supporting Information

Coherence in the Ferroelectric A_3ClO (A = Li,Na) Family of Electrolytes

Maria Helena Braga

LAETA-INEGI, University of Porto, Engineering Faculty, Engineering Physics Department, R. Dr. Roberto Frias s/n, 4200-465 Porto, Portugal; mbraga@fe.up.pt

Graphs in Figure 2d can be obtained using wxMaxima 20.06.6, the Runge-Kutta method and the macros:

x vs time:

```
(%i1)   eqs: [y,-x+5*(1-x^2)*y-50*sin(7*t),1]$
(%i2)   v: [x,y,t]$
(%i3)   v0: [0.001,0,0]$
(%i4)   p: rk(eqs,v,v0,[t,0,50,0.01])$
(%i5)   last(p);
(%o5)   [50.0,-
2.102520981419923,1.654017020965303,49.99999999999862]
(%i6)   plot2d ([discrete,makelist([s[1],s[2]],s,p)],
    [xlabel,"t"],[ylabel,"x(t)"],[legend,false],[nticks,30]);
```

Phase portrait:

```
(%i1)   eqs: [y,-x+5*(1-x^2)*y-50*sin(7*t),1]$
(%i2)   v: [x,y,t]$
(%i3)   v0: [0.001,0,0]$
(%i4)   p: rk(eqs,v,v0,[t,0,50,0.01])$
(%i5)   last(p);
(%o5)   [50.0,-
2.102520981419923,1.654017020965303,49.99999999999862]
(%i6)   plot2d ([discrete,makelist([s[2],s[3]],s,p)],
    [xlabel,"x(t)"],[ylabel,"y(t)"],[legend,false],[nticks,30]);
```

References:

1. Maxima software https://maxima.sourceforge.io/ (Accessed on 04.05.2021).
2. Jaime Villate. *Dinâmica e Sistemas Dinâmicos*. 2019 https://books.fe.up.pt/index.php/feup/catalog/book/978-972-99396-5-5 (Accessed on 04.05.2021).
